# Patients as qualitative data analysts: Developing a method for a process evaluation of the ‘Improving the Safety and Continuity of Medicines management at care Transitions’ (ISCOMAT) cluster randomised control trial

**DOI:** 10.1111/hex.13257

**Published:** 2021-05-01

**Authors:** Catherine Powell, Hanif Ismail, Richard Cleverley, Andrew Taylor, Liz Breen, Beth Fylan, Sarah L Alderson, David P Alldred, the ISCOMAT Programme Team

**Affiliations:** ^1^ School of Pharmacy and Medical Sciences University of Bradford Bradford UK; ^2^ Wolfson Centre for Applied Health Research Bradford UK; ^3^ Patient and Public Involvement Representative ISCOMAT Patient‐Led Steering Group Bradford UK; ^4^ NIHR Yorkshire and Humber Patient Safety Translational Research Centre Bradford Institute for Health Research Bradford UK; ^5^ Leeds Institute of Health Sciences University of Leeds Leeds UK; ^6^ School of Healthcare University of Leeds Leeds UK

**Keywords:** patient participation, qualitative research, research design

## Abstract

**Background:**

How to meaningfully partner with patients as data analysts remains obscure. A process evaluation of the ‘Improving the Safety and Continuity Of Medicines management at care Transitions’ (ISCOMAT) cluster randomised control trial of an intervention for improving medicines use for people living with heart failure is being conducted. The intervention includes patient held information on heart medicines and care, enhanced communication between hospital and community pharmacists, and increased engagement of community pharmacists with patient care post‐hospital discharge. ISCOMAT patients living with heart failure were interviewed about experiences with the intervention. We sought to gain insights from patients on data collected to enhance our understanding of experiences with the intervention.

**Objective:**

To develop a method for involving patients as analysts of qualitative data in a process evaluation.

**Design:**

Patients and researchers co‐analysed qualitative data. A framework method was applied involving; familiarisation, coding, developing an analytical framework and interpretation. The process was facilitated through home working and a workshop with a training component.

**Results:**

The co‐designed framework enabled researchers to map all further patient interview data. Patients' specialist knowledge enhanced understanding of how the ISCOMAT intervention can be best implemented.

**Conclusions:**

Patients’ unique experiences can enhance validity and rigour in data analysis through sharing their interpretations of qualitative data. The involvement process is crucial in elucidating knowledge and avoiding tokenism. As analysts, patients gain an appreciation of research processes, building trust between researchers and patients. Group dynamics and involving patients throughout the whole research process are important considerations.

## 1 INTRODUCTION

Patients are increasingly involved in health research. Research funders both require and support patient public involvement (PPI) in research.^1^, ^2^ While patients are involved in phases of research, from conception through to dissemination,^3^ only a limited number of studies involve patients beyond an advisory capacity and as co‐researchers.^4^, ^5^, ^6^ Where co‐researcher studies have been conducted, the benefits to science have been under‐reported. The scientific value of patient involvement needs to be highlighted to avoid a tokenistic approach.^7^ Patients can add 'experiential knowledge', personal insights of how individuals cope and live with illness or disability. PPI in data analysis could improve the relevance, focus and real‐world acceptability of health research;^8^ however, the process for this remains obscure.

'Equity' has been a key concern underpinning approaches to PPI in health research.^9^ PPI has the potential to empower individuals and communities to have a significant role in shaping health and social care research. For example, participatory research has sought to redress power imbalances for indigenous minority populations. By democratising health and social care research, we can realise its potential to provide maximum health and social benefits.^2^, ^10^ The generation of shared 'knowledge spaces' where patients and researchers with different perspectives can interact could form a vital part of a co‐analysis process.^5^, ^11^ Patients require 'appropriate' knowledge and skills to effectively carry out their role.^12^ Training that is flexible according to skills, interests, topic and project type is important in ensuring meaningful PPI.^13^ Training would need to avoid ‘professionalising’ patient viewpoints, enabling authentic patient views to enhance an understanding of research as opposed to encouraging views from an academic perspective. ^14^ Practical considerations must be considered, such as the ability to travel to attend meetings.^15^

Patients’ capacity for involvement may vary, and projects have resource limitations.^9^ Given these considerations, how can patients be meaningfully involved as analysts to enhance scientific value? We sought to involve patients as analysts for our process evaluation of the Improving the Safety and Continuity Of Medicines management at care Transitions (ISCOMAT) cluster randomised control trial. The co‐designed ISCOMAT Medicines at Transitions Intervention (MaTI) seeks to make best use of medicines and reduce harm through: patient held information; enhanced communication between hospital and the patient's community pharmacist; and increased engagement of the community pharmacist post‐hospital discharge.^16^ The process evaluation collected interview data from ISCOMAT patients on their experiences of the intervention. We aimed to develop a method of meaningfully involving patients as analysts of patient interviews in a process evaluation.

## 2 METHODS

### 2.1 The ISCOMAT process evaluation

**FIGURE 1:**
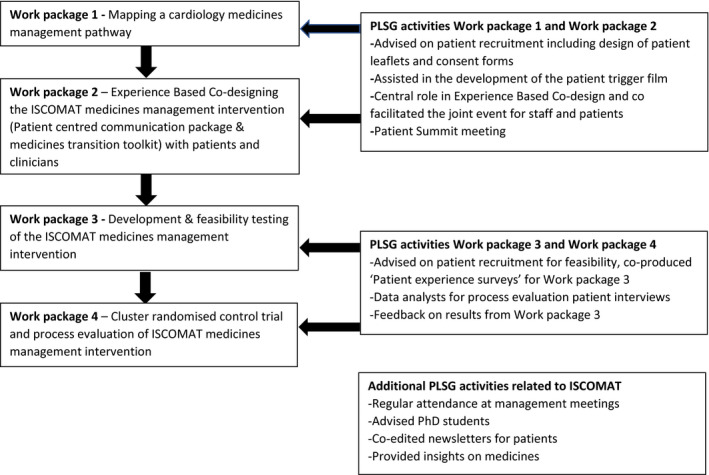
ISCOMAT Work packages and PLSG activities

The ISCOMAT programme began in 2016 and involves four work packages to develop and test an intervention in a cluster randomised control trial, to improve medicines management for patients transferring from hospital to community. The experience‐based co‐designed intervention involves the use of patient held information, enhanced communication between the hospital and the patients' community pharmacists and increased engagement of primary care staff after discharge for heart failure patients.^17^ Further detail on the intervention and results will be provided in later papers. The ISCOMAT process evaluation seeks to inform interpretation of the trial findings, inform implementation of the intervention on a wider scale and aid dissemination of the intervention. The process evaluation involves collation of multiple sources of data, such as field notes of hospital observations and interviews with health professionals and patients from six research sites. Two researchers interviewed 20 patients across England who live with heart failure to understand their experiences of the intervention. Further details of the process evaluation are published in a protocol paper.^18^ The researchers invited the ISCOMAT patient‐led steering group (PLSG) to partner with researchers in co‐analysing these patient interviews. Figure 1 illustrates PLSG involvement across the four work packages of ISCOMAT.

### 2.2 The analysis team

The analysis team were two researchers, four wider process evaluation team members and five patients who were members of the PLSG. One patient took part in the early phases but was unable to continue due to deteriorating ill health. Patients were identified and recruited through local networks or following co‐design groups in work package two where they subsequently agreed to join the PLSG. Four members were living with heart failure and one was a carer. Two patients had degree‐level education while others had diploma‐level education. Two patients were in full‐time employment. One additional patient who later joined the PLSG was involved in the later phase of interpretation only. Ages ranged from 32 to 84. Most analysts were male with one female in the group. Patients’ experience in research prior to ISCOMAT was varied with some being involved in other projects. None had been involved in qualitative data analysis. The two researchers facilitating the meeting had previous experience with PPI and conducting qualitative research.

The ‘patient analysts’ (PLSG involved in analysis) were involved in ISCOMAT primarily in an advisory capacity from inception to present trial phase. Patients’ analysts developed the interview topic guide, although they were unable to recall specific details. PLSG meetings are held quarterly, with on‐going communication between researchers and PLSG members through phone and email. Interactions are research focused and social, helping the group to build strong relationships. This was the first time patients were involved in qualitative data analysis in ISCOMAT or any other capacity. One training session was provided as part of the analysis process. No other training had occurred prior to the PLSG becoming analysts.

### 2.3 Qualitative analysis method

A framework approach to qualitative data analysis was applied. Framework analysis, originally, created by Jane Ritchie and Liz Spencer,^19^ is a frequently used qualitative analysis method in UK health research.^20^ The framework approach involves identifying a framework, indexing, charting, and mapping and interpretation.^19^ Gale et al^20^ later developed seven key steps to carry out these tasks, ‘Step one Transcription', creating an audio and verbatim transcript; 'Step two Familiarisation with the interview', reading and reflecting on a full interview transcript; 'Step three Coding', rereading the transcript and applying labels to important phrases; 'Step four Developing a working analytical framework', the team agrees on a set of codes for all subsequent transcripts; 'Step five Applying the analytical framework', indexing using existing coding; 'Step six Charting data into a framework matrix', summarising data from categories into a matrix and 'Step seven Interpreting the data', an iterative process including exploring connections between categories and interrogating theories. The process recommends the involvement of lay members in the analysis process yet does not provide detailed guidance on how to accomplish this.^20^

Our analysis occurred alongside data collection. The first five interview transcripts (of a final 20) were selected based on a range of intervention sites, gender, age, length of diagnosis and fidelity to intervention. The five patient analysts were given one unique anonymised transcript. Alongside the transcript, patient analysts were instructed how to familiarise themselves with qualitative data, involving reading interviews and noting in the margin anything of interest concerning the intervention only. An example annotated transcript was provided. Patients were asked to bring and prepare a summary of their transcript at an analysis workshop. Framework analysis was selected as an appropriate method for involving patients. Evidence for a particular qualitative method, involving patients as analysts and for use in evaluating a health intervention, was limited. However, Framework analysis is known for its strengths in facilitating the involvement of less experienced researchers, and involving multiple individuals at multiple stages.^20^ Developing a process of integrating patients' expertise with Framework analysis could improve validity and rigour.

Reflections on the data analysis process were supported through feedback forms, on‐going discussions, an audio recording of the workshop, as well as authoring this paper. Feedback forms enabled PLSG analysts to reflect on the training and workshop day considering: the most helpful parts of the workshop and training, whether to take part in future qualitative data analysis, key contributions made, and suggested improvements.

### 2.4 Ethics

The ISCOMAT trial and process evaluation were approved by Research Ethics Committee and the UK Health Research Authority REC: 18/YH/0017 / IRAS: 231431. It was not necessary to gain additional ethics committee approval specifically to involve patients as analysts. The PLSG had no contact with the interviewees or access to identifiable data. The PLSG agreed to terms of reference in June 2017. Informed by INVOLVE guidelines,^21^ the terms of reference outlines that the patient's role will be to contribute to development and be accountable to the leaders of the research project. Some activities include contributing to reviews and evaluations of the ISCOMAT programme, advising on dissemination and providing patient perspective on research deliberations. The researchers checked with PLSG members whether they would like to take part specifically as analysts. The ISCOMAT patient payment policy is informed by INVOLVE guidelines^22^ and is as follows: £110 per meeting for attending Steering/Management Group meetings, £75 for a co‐design event or meeting, £75 for attending other training events, travel costs, £20 per hour for additional work for co‐analysis event. Patients gave their permission for this paper to be written, including quotes. The PLSG agreed on two members to work on this paper. These two co‐authors (RC and AT) have made an important contribution, regularly meeting with researchers (CP and HI) to revise the manuscript.

## 3 RESULTS

**FIGURE 2:**
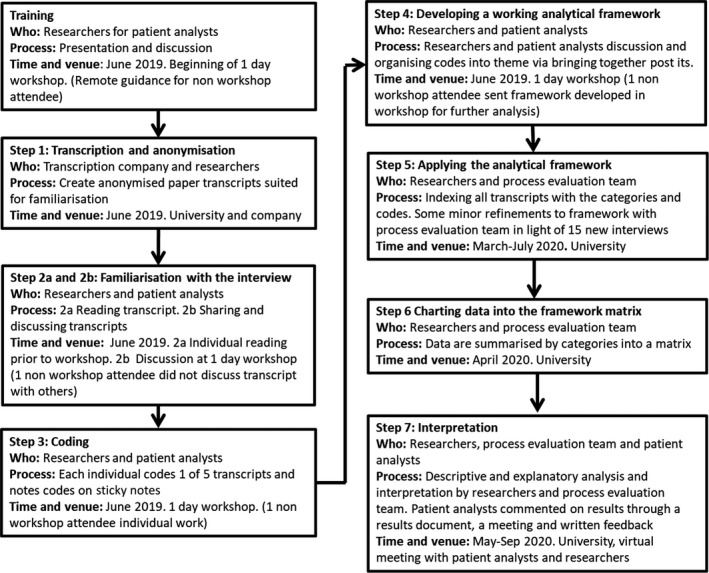
Co‐analysis method and process of applying Framework analysis

We report on the process by which patient analysts were involved in the analysis. Figure 2 illustrates the steps in the process.

### 3.1 Training

Patient analysts required appropriate knowledge and skills to complete the analysis. A one‐day face‐to‐face analysis workshop began with training delivered by the two researchers. Four patient analysts attended the workshop and training. One patient analyst was unable to attend on the day.

Patients were enthusiastic to engage with interview data as opposed to purely the method itself as they had personal interest as heart failure patients. As one patient highlights, ‘Being part of the ISCOMAT team has been one of the best experiences of my life. Being a patient with a long‐term health condition can often make you introverted, but knowing that there are researchers out there running studies, that could potentially change patients’ lives for the better, makes patients want to join in and give their knowledge’.

The training provided an outline of thematic analysis, along with the importance of coding and how this process is undertaken. A presentation was given by the researchers. The researchers encouraged patient analysts to focus on the interview content, key research questions and the topic guide in their analysis, with sufficient opportunity provided for questions and discussion. The researchers encouraged patient analysts to engage with the content rather than critiquing the data collection process. This was necessary as patient analysts were more familiar with advising on study processes rather than engaging in data analysis .

The training was well‐paced and helped patient analysts to develop an understanding of the analysis process. One patient analyst felt ‘[the training] helped me understand what was expected of me during the analysis/workshop. It helped give me an understanding of what to do with the notes I had made on the transcripts and also built up my confidence in the subject. [The researcher] took her time and went through each detail answering any questions we may have had and stopping to check we were all following. I did not feel left behind’.

Another patient analyst felt the most important part of the workshop was ‘understanding what the term meant. The process is like squeezing a lemon. You get the last drop out of data from…what you might think is disconnected information’. Another patient describes how they were able to analyse.

‘Starting the coding was difficult at first. I was worried I would make a mistake, but once I got the first few written and logged, the task became much easier and more enjoyable. I found coding the transcripts really simple once I built up the confidence to make a start. I soon realised that my experience and knowledge of being a long‐term patient made it easier for me to pick out sections of the transcript that felt the most valuable to the project’.

Thus, this new knowledge enabled patient analysts to understand how conclusions are drawn from interview data.

### 3.2 Framework analysis of patient interviews process

Below, we describe the analysis process. We have adhered to the seven‐step process in our analysis,^20^ developing a method to involve patients in earlier steps two‐four, and step seven.

Step one: Transcription and anonymisation.

Researchers checked the transcripts to ensure anonymity and presentation requirements were accurate. Transcripts were accessible (text size, sufficient margins) to aid analysis.

Step two (a): Familiarisation with the interview: individual work before the workshop.

Providing one transcript allowed patients to have sufficient time to fully immerse themselves in an unseen interview. Researchers familiarised themselves with all transcripts given their prior research knowledge and skills, involvement in data collection and need for oversight to facilitate the workshop. One patient reflects on their increased confidence in the process.

‘When we received the transcripts I was nervous as to what was expected of us. Reading through the transcript I felt better about the task at hand as it was easy to relate to the patient’s experiences’.

Step two (b): Familiarisation with the interview: the workshop.

To give patient analysts insight into the remaining unread four transcripts, each provided a verbal summary of their annotated transcripts to the group during the data analysis workshop held at the university. They described key areas of an interview and spoke about how the transcript resonated with their own experiences or those they have heard about, providing a unique contextual understanding of data. Patient analysts and researchers were enthusiastic to learn about interpretations from other patient analysts. One patient analyst was unable to attend the workshop and therefore did not gain this additional insight. The researchers reflected that some patient analysts appeared surprised by the transcripts, being unfamiliar with reading individuals’ words written verbatim. Thus, giving patient analysts sufficient time in advance to read the transcript worked well in enabling familiarisation with the content and the organisation of interviews into verbatim transcripts.

Step three: Coding.

Following the discussion on transcript summaries, each group member coded their interview transcript. Patient analysts were asked to reread their transcript and focus coding in three key descriptive categories relevant to interviewee experiences of the intervention. They were asked to write the codes on sticky notes, identifying the transcript and line numbers.

The process of moving from step two to coding, where specific data are extracted with a focus on key research aims and objectives, had been described in the training. The researchers provided more support during the coding process to ensure that patients were able to effectively make this transition. One action involved keeping the research aims and objectives in view. The researchers noticed how patient analysts were able to recognise barriers and facilitators to the ISCOMAT intervention. Being able to relate to interviewees as heart failure patients and carers, and their knowledge of ISCOMAT were key factors in facilitating this process. For example, one aim was to identify barriers and facilitators to experiences with community pharmacy. In the extract below, a patient describes their lack of knowledge and communication with community pharmacy. The patient analyst was able to code this as ‘negative points re medication and pharmacy’ once they considered barriers they had when accessing community pharmacy themselves.

’R: Yes well I didn't even know they had a consultation room.

I: All these little rooms in the corner where you can go in and have a chat in privacy.

R: I mean I’d never heard of a community pharmacy office sort of thing. I thought, you know, all they did was dish out drugs. I didn't know that they actually do the conditions themselves’.

Patients analysts valued the knowledge and skills developed through the coding process. In written feedback one patient mentioned, ’with [sticky] notes, we then proceeded to flag all the parts of the transcripts we found the most important…It enabled me to improve upon my skills of analysing…and develop the new skill of coding’. Through understanding and applying the newly acquired skill of coding, patient analysts had a method of communicating their interpretation of data.

Step four: Developing a working analytical framework.

The sticky notes with coding were placed on papers organised by the three key categories: experiences at community pharmacy, experience with medicines and experience with the patient held information. The patient analysts and researchers discussed and identified themes from the coding, identifying repeated ideas and grouping them with a common point of reference. In addition to the training, group working supported patient analysts understanding of how to move from codes to developing a thematic framework. For example, one theme around ‘communication’ was created as a barrier to experiences with the pharmacist. Sticky notes with coding relevant to this agreed theme were placed underneath. The example provided earlier of a patient coding ‘negative points re medication and pharmacy’ was placed under this ‘communication’ theme. Other examples include, ‘confusing messaging between GP/community pharmacy’, and ‘no existing relationship with pharmacist’. Some themes and coding under barriers to use of the patient held information included ‘too much information’, ‘burdensome to complete pull out [section of the patient held information], and ‘timing of [patient held information being delivered].

Some patients found this process more challenging. Sharing knowledge facilitated the framework development process. Patient analysts were able to ‘*interchange…ideas and observations’*. Researchers were able to facilitate discussions and convey how to create themes. One patient analyst felt comparing interpretations with older patient analysts was extremely interesting.

‘When asked to divide our findings into categories I was eager to start. I had several [sticky notes] that I thought should go in particular categories, but as I stepped back and looked at what others had done I realised there were many angles from which to interpret this data. It was good to hear the thoughts and reasonings of the other members of the group. We were able to debate and have our suggestions accepted or rejected in a friendly and lively discussion. Listening to other perspectives and points of view has helped me to appreciate the many ways in which data can be described’.

The process enabled patient analysts to get close to data, and co‐construct themes across multiple interviews based on their knowledge from lived experiences of heart failure. Through co‐developing the framework as a group, key themes were elucidated in the discussions between patient analysts and researchers. Crucially, patient analysts’ interpretations enhanced the framework. Themes reflected the patients’ in‐depth understanding of living with heart failure, embedding interview data within the wider experience. This helped researchers to understand the key reasons for patient interviewee experiences of the ISCOMAT intervention.

For the patient analyst unable to attend the workshop due to time and capacity limitations, the researchers organised a system to support their involvement. The patient analyst was sent: the framework developed in the workshop organised into a matrix, written instructions, and a transcript. The instructions prompted the patient analyst to decide whether the framework ‘made sense’ and whether they could identify new themes. An example was provided, and they were asked to read the transcript searching for quotes, mapping them onto the matrix and leaving cells blank where no relevant quote appeared.

Without group interaction and training, this patient had difficulty understanding how the framework could be developed from transcripts.

‘When I embarked on the coding of the transcripts, I initially found it quite difficult to know where to start. Although there were some basic instructions of what was being looked for, it was hard to know how to approach the task when faced with the transcript and code side by side. I initially read through the coding to get an idea of the areas in the transcript I was looking for and then read through the transcript to see if the coding areas related to specific sections of the coding. It soon became apparent that this wasn’t the case, so I then went back and tried to familiarise myself more closely with the individual elements in the coding so I could identify when lines within the transcript matched with them. I would then write the lines of the transcript next to the related coding elements. I needed to repeat this a few times in order to feel confident that I hadn’t missed anything out’.

Through a process of contrasting between transcript and framework, it was eventually possible to code. Modifying the predeveloped framework according to the transcript posed difficulties. The patient was unclear as to whether they could make changes. The patient coded the transcript and mapped on quotes, not making any alterations to the framework developed in the workshop. Researchers' reflected that they had held back on providing remote training out of concern of burdening the patient analyst who was working remotely with several documents, yet training would have enhanced the patient's ability to analyse data.

A framework for analysing our patient data was created, identifying key barriers and enablers to the experiences at community pharmacy, experience with medicines, and experience with the patient held information. It was crucial in organising data and enabling researchers to develop further interpretations. Such knowledge helped in developing a critical understanding of interviewees experiences with the intervention.

Step five: Applying the analytical framework.

The researchers analysed the remaining 15 patient transcripts. All 20 interviews were indexed according to the co‐analysed framework. Qualitative data analysis software NVivo 12^23^ was used for this process. The researchers felt the co‐developed framework worked well. Only minor alterations to the framework were made in light of new transcripts and internal discussions. Patient analysts were not involved in step five.

Step six: Charting data into the framework matrix.

The researchers summarised the 20 transcripts according to key themes identified. The summaries were displayed in a matrix using NVivo 12.^23^ The display facilitated the interpretation of the data at step seven, as well as enabling the researchers to rapidly make connections between the summaries and the original coded transcripts. The process evaluation team checked the quality and consistency of the summaries. Patient analysts were not involved in step six.

Step seven: Interpretation.

Researchers, the wider process evaluation team and patient analysts were involved in step seven. The researchers conducted descriptive and explanatory analysis. Patient analysts commented on the interpretations. This process enhanced the trustworthiness of findings, through establishing the transferability of data.^24^ Due to the restrictions imposed by COVID‐19, patient analysts and researchers could not meet face to face. Virtual small group and individual meetings were organised via phone and a preferred online platform (Microsoft Teams). Patient analysts had met with researchers remotely for regular meetings prior to the interpretation. One patient who had a technical difficulty was supported through discussions about how to use the technology; however, patient analysts had IT literacy. An interpretation document was circulated in advance. With permission, discussions were recorded, and written notes taken. Further written feedback was provided via email, enabling reflection before, during and after the discussion. Patient analysts commented on the findings, mapping their own experiences on to the analysis. These insights contextualised the interpretation, giving a deeper nuanced meaning. The experience helped patient analysts understand the full process of qualitative analysis.

## 4 DISCUSSION

We developed a process for involving patients as qualitative analysts. A framework was co‐designed, which enabled researchers to index and interpret all further interview data. Patient analysts checked the trustworthiness of the interpretations, a process advocated by Lincoln and Guba (1985).^24^ The process followed the stages of framework analysis, with patients involved in familiarisation, coding, creating a framework and interpretation. Group working was crucial for co‐designing the framework.

The scientific value of patient involvement in research has often been overshadowed, with a focus on patients' experiences.^7^ We have identified the value of patient analysts for a process evaluation of a large‐scale cluster randomised control trial of a health‐care intervention. Patient analysts were able to enhance researchers understanding of how the intervention was experienced, providing vital context. This was achieved through moving beyond surface level explanations, focusing on detail and context, generating meaning from the interview transcripts.^25^ The process brought patient analysts closer to data, prior to interpretations. The process evaluation, aimed to consider intervention implementation, and how it could be used and sustained across different settings. Patient analysts enhanced this understanding.

Sustaining relationships between patients and researchers throughout a project can support patient involvement. Hoddinott^26^ highlights the distinction between ‘partnership’, ‘involvement’ and ‘participating’, where ‘participants’ are the subject of the research, those ‘involved’ work with researchers or in ‘partnership’ as having an on‐going relationship with trust respect and an equal voice. Debates that took place in the workshop were facilitated due to existing relationships, while also enhancing them. Moreover, patients had in‐depth knowledge of ISCOMAT having been involved in it previously, enabling them to focus purely on the data analysis process. Building up relationships and trust at the earlier phases of research projects can make a critical difference to the analysis process. Group working and existing relationships prevented a tokenistic approach.

PPI needs to be flexible to each research project.^13^ Patients can be upskilled in research through relevant training, supporting their meaningful involvement.^12^ Our face‐to‐face approach enabled patients to code data and develop a framework with a relatively short training component. Patient analysts’ and researchers’ knowledge was shared through the process. The framework method enabled multiple and less research experienced analysts to code data.^20^ Some projects have developed more intensive methods for patient analysts in their analysis process, through for example, running multiple workshops.^5^ However, such an intensive approach may not be appropriate for patients involved in all projects. Involvement needs to consider appropriateness for patients, ensuring they can contribute how and when they choose to do so.^27^ Enabling patients to be involved in a meaningful way in large‐scale projects is crucial. However, there is limited research that has considered how to effectively involve patients as co‐analysts in a complex process evaluation of a large‐scale cluster randomised control trial. The ISCOMAT patients living with heart failure, who may not be well enough on any given day, or have other commitments, may have had difficulty in committing to a more intensive process. The process we developed reflected patients’ needs, the research design of the ISCOMAT programme, and was adapted due to COVID‐19. Patient analysts were able to continue the process remotely given their relatively high educational background and IT literacy. However, patient analysts stressed that they did not have previous qualitative analysis experience, nor academic backgrounds. Replicating this analysis process may not be possible with those with low educational background, and/or lacking access to and ability with information technology.

A key strength has been the enhancement of our patient analysis, with the co‐designed framework informing all further patient data analysis. All patients valued the opportunity to become analysts. One patient commented that, ‘personally, I feel that this experience was the most interesting and rewarding part of the study…I would very much like to be part of workshops like this in future projects’. However, our process had limitations. The one‐day training and workshop was tiring for some. The team could have explored ways of facilitating patients to develop the mindset for analysis prior to a workshop. Training could have been provided through a recording of the initial presentation, group working and meeting together after. Researchers felt that the training needed to occur before the coding process and were concerned with burdening patients. Any concerns about burdening patients, however, should be discussed with patients themselves. For example, researchers held back on sending extensive information for the patient analyst working remotely as they felt too much information could be onerous. However, this patient analyst felt they needed more information to analyse the data. They also felt they would have gained more from group interaction and would have understood more of the analysis process. We would therefore recommend creating at least some opportunity for group members to interact, particularly in developing a framework. Methods could perhaps explore asynchronous group interactions if patients are unable to meet at one time. The patient analyst working remotely suggested holding a group plenary session with other patient analysts and researchers, after coding. This would have provided validation that they had completed the exercise correctly. The researchers felt that discussing analysis process plans more in advance could have improved patient analysts experience. However, patient analysts were satisfied with their involvement in analysis plans.

## 5 CONCLUSION

We have developed a method for involving patients as analysts of qualitative data in a process evaluation. The method involved a combination of a one‐day workshop involving a short training component and home working. The process enabled key themes to be co‐constructed in discussions between patient analysts and researchers, creating a framework for the analysis of all further patient data. The approach taken enabled the process evaluation team to learn from patients to gain a deeper understanding of the implementation and transferability of an intervention. We were able to learn what are the barriers and facilitators to use of patient held information, experiences with community pharmacy and medicines experience.

We suggest the process as an example for analysis of qualitative data in complex process evaluations of clinical trials. Developing a rigorous process which meaningfully involves patient analysts is crucial for the research to hold scientific value. Key considerations around patient and researcher group dynamics, and research design needs to be made clear when selecting the process. Researchers should not only contact patients when they require help. Relationships between researchers and patients need to be built prior to an analysis process, with constant engagement, keeping up to date and developing rapport throughout the project's lifespan. Shorter projects would need to develop methods to build relationships in a limited timescale; which might be achieved through more meetings, emails and communication.

## 6 DISCLAIMER

The views expressed are those of the authors and not necessarily those of the NIHR or the Department of Health and Social Care.

## 7 PATIENT CONTRIBUTION

Patients contributed as co‐analysts and as co‐authors.

## CONFLICT OF INTEREST

We are not aware of any conflicts of interest.

## AUTHOR CONTRIBUTION

All authors revised the manuscript critically for important intellectual content and made substantial contribution to conception and design. Catherine Powell and Hanif Ismail drafted the manuscript. All authors have given final approval of the version to be published. Each author has participated sufficiently in the work to take public responsibility for appropriate portions of the content and agreed to be accountable for all aspects of the work in ensuring that questions related to the accuracy or integrity of any part of the work are appropriately investigated and resolved.

## Data Availability

Data sharing not applicable to this article as no datasets were generated or analysed during the current study.
